# A qualitative exploration of the impact of COVID-19 on food decisions of economically disadvantaged families in Northern Ireland

**DOI:** 10.1186/s12889-021-12307-1

**Published:** 2021-12-16

**Authors:** Eleni Spyreli, Michelle C. McKinley, Jayne V. Woodside, Colette Kelly

**Affiliations:** 1 Centre for Public Health, Queen’s University Belfast, Belfast, BT12 6BA UK; 2 Institute for Global Food Security, Queen’s University Belfast, Belfast, BT9 5DL UK; 3 Health Promotion Research Centre, School of Health Sciences, NUI Galway, Galway, Ireland

**Keywords:** COVID-19 lockdown, Food decisions, Low-income families

## Abstract

**Background:**

The first UK-wide lockdown to prevent the spread of COVID-19 had a serious financial impact on low-income households, a population already in higher risk of food insecurity and poor dietary choices. Qualitative data on the impact of COVID-19 lockdown on food decisions of UK families are scarce. This study aimed to explore how the measures to control the spread of COVID-19 influenced the food-related decisions of socioeconomically deprived families in Northern Ireland.

**Methods:**

A qualitative study captured data from online individual interviews. Participation was open for parents of children 2–17 years old living on a tight budget in urban and rural areas of Northern Ireland. A sampling matrix enabled equal representation of single- and two-parent households, as well as parents of younger children (<12y) and adolescents (≥12y). Data were collected by using the methods of Photovoice and mapping exercise. Data were analysed through a thematic approach.

**Results:**

Twelve online interviews were conducted and five distinct themes were identified reflecting families’ food-related decisions that were affected by the COVID-19 lockdown: 1) food planning; 2) food purchasing; 3) meal preparation; 4) eating and feeding behaviours and 5) eating food prepared outside the house.

**Conclusions:**

The restrictions put in place to inhibit the spread of COVID-19 influenced all aspects of dietary decisions of low-income families. Changes observed during this period included frequent consumption of homemade meals, but also increased unhealthy snacking. Infrequent food shopping encouraged good meal planning, but was also a barrier to securing adequate fresh food. Food-related support including school meal assistance contributed to families’ food security, particularly those of single parents.

**Supplementary Information:**

The online version contains supplementary material available at 10.1186/s12889-021-12307-1.

## Background

In the UK since the first cases of COVID-19 infection were identified in late January 2020 [1], the outbreak of COVID-19 has escalated into a major public health crisis [2]. The nationwide lockdown, enforced at the end of March 2020 in response to the virus outbreak, involved the closure of ‘non-essential’ businesses, cessation of social events and social-distancing measures [3–6]. Vulnerable groups with underlying conditions were also encouraged to shield at home.

Apart from the serious health implications, the COVID-19 pandemic had an impact at a social and economic level. In a survey of non-COVID patients living in England, Wales and Scotland various sources of anxiety were mentioned including: uncertainty regarding finances and employment; and access to and availability of food [7]. In June 2020 in the UK around 300,000 people were removed from their post because of the COVID-19 pandemic and received no pay [8]. These individuals were more likely to be in occupations that require a minimum level of education. This means that households of individuals with lower educational attainment and income were very likely to be financially burdened by lockdown.

To fully understand the implications of COVID-19 for low-income households, one also needs to consider that the economically disadvantaged families are also more likely to have a lower quality diet [9–11], which in turn, renders them vulnerable to the COVID-19 virus [12]. Inequalities in nutritional intake due to income are well reported in the literature and socioeconomic deprivation is associated with energy-dense, nutrient-poor diets. In Northern Ireland particularly, where intakes of total and saturated fat are already increased compared to the rest of the UK, low-income households tend to consume fewer fruit and vegetables and have lower intakes of dietary fibre and some vitamins and minerals when compared to higher-income families [13, 14]. Specifically for children, there is a significant increase in intake of total fat and high-sugar drinks and snacks with decreasing household income [14]. Habitual diets high in energy, saturated fats and refined carbohydrates contribute to the prevalence of obesity and type-2 diabetes [15], both of which are significant risk factors of COVID-19 pathology and mortality [16, 17]. Additionally, considering the limited access to fresh food due to shielding and food insecurity often experienced by economically disadvantaged households [18, 19], the period of the pandemic has potentially further exacerbated the nutritional problems experienced by these families.

A substantial amount of research has been published about the effects of the COVID-19 pandemic on food-related decisions (food planning and purchasing, meal preparation) in the UK and globally with most of the existing data being quantitative rather than qualitative [20–23]. Qualitative data provide a rich description of individuals’ perceptions and experiences, such as those in relation to health behaviours and eating habits [24]. Therefore, employing a qualitative methodology is highly appropriate to explore how COVID-19 and the measures to control its spread influenced the food decisions of low-income households.

The objective of this study was to explore the impact of COVID-19 lockdown on food-related decisions as experienced by economically disadvantaged families in Northern Ireland. Gaining a good understanding of the nutritional challenges that low-income families faced during the first nationwide lockdown (Mar – Jul 2020 [25]) is vital to develop appropriate interventions to meet their needs in the aftermath of the pandemic.

## Methods

The present qualitative study arose from a larger project that aimed to explore the environmental factors that drive food choices in low-income families with children on the island of Ireland. Data presented in this paper however, have been collected exclusively in Northern Ireland.

Northern Ireland, the smallest of the four UK countries, has a predominantly white population (98% based on 2011 census data) [26]; and even though it is possible that the area has become more ethnically diverse since 2011, the current proportion of non-white residents is still expected to be very low. The number of people in poverty in Northern Ireland has significantly fallen in the past decade [27]; however, the proportion of children living in low-income families remains high, with this proportion being one out of three in some parts of the country [28].

Ethical approval for this study was obtained by the Research Ethics Committee of Faculty of Medicine, Health & Life Sciences, Queen’s University Belfast.

### Participant selection

Participation was open to parents or guardians of children in preschool, primary and post-primary school age (2–17 years), as specified by the original project protocol. Both mothers and fathers were invited to take part. Recruitment focused on families that self-identified as living on a tight budget and lived in rural and urban areas in Northern Ireland. All participants needed to own a smartphone to be able to engage in the Photovoice and mapping exercise. Families with children who had a clinical diagnosis affecting eating (e.g. gastrointestinal conditions or diabetes) were excluded, as their experiences regarding food decisions during the COVID-19 lockdown would differ from the families of interest for this study. Families with children with well-managed food allergies were included.

The sample size was determined a priori with parents from 12 distinct households required. This number was deemed necessary to ensure different types of families could be included. A stratification process was followed so that the final sample included equal numbers of single parents and parents living with a partner, as well as equal numbers of parents with children 2–12 and 12–17 years old.

As part of the recruitment process the research team utilised a list of known contacts (i.e. gatekeepers) who worked in community organisations in Northern Ireland and interacted with low-income families. The gatekeepers extended the call for recruitment to their service users by posting in social media or forwarding the study information to further gatekeepers who worked with the target population. All parents who came forward were asked to confirm whether they were on a tight budget during the lockdown.

### Data collection

A first one-to-one discussion was conducted by phone to describe the purpose of the study and what participation required and capture participant’s demographic information. Data collection methods were as follows:

#### Photovoice

For this study, participants were instructed to take photographs that represent factors influencing their food choices during the COVID-19 pandemic and present these photographs during the online interviews. Participants could capture images of food promotions (offers and advertisements), food shopping (shopping lists, receipts, shopping bags), meal preparation (food being cooked/served), or anything else they felt impacted their food decisions. They were encouraged to take 1–2 photos with their phone every day for 1 week and were also given the option of receiving a reminder text. They were also advised to avoid taking identifiable images of people.

Photovoice gives participants the opportunity to capture their own images and through them record their environment and share their personal experiences [29]; experiences that are often under-represented in research due to its difficulty to ‘reach’ low-income settings. Moreover, by ‘putting’ cameras in the hands of participants Photovoice reduces the power differential between researcher and participant [30]. Photovoice has been a valuable data collection method to understand individuals’ dietary experiences [30, 31], as well as for studies using remote methods during the pandemic [32, 33].

#### Mapping exercise

To complement Photovoice, an additional interactive approach was utilised. Participants were invited to draw their local food environment and to include shops they use and do not use. These included supermarkets, small food retailers, restaurants and take-away shops. In this way, parents could depict their local area, draw attention on particular features and places and elaborate on the relationships between these places and their food decisions [34].

After giving detailed guidance on the above methods a follow-up interview was arranged for the following week based on families’ availability. Participants were instructed to share their photographs and map with the research team via email prior to the interview. Interviews were conducted online through the *Teams* software (Microsoft Corporation, Redmond, Washington, US). All photographs submitted included content about food and were discussed during the interview. Additionally, the map was used to discuss the strategies families employed to navigate their food environment and how the local food environment influenced their food choices during lockdown. A topic guide was used to guide the photo-elicited interviews (see Supplementary File). All interviews took place in June 2020 and were moderated by a researcher (ES, dietitian) with previous experience in qualitative interviews. All participants consented to being included in the study by signing a form which was emailed to them. As a token of appreciation for their time participants received a £35 *One-4-all* shopping voucher.

### Data analysis

Interviews were recorded and transcribed verbatim with the relevant functions offered by *Teams*. Transcripts were checked for inaccuracies and to gain familiarity with the data. A thematic analysis approach was used, as described by Braun & Clark [35]. A researcher (ES) read all transcripts line-by-line and allocated codes without trying to impose a pre-existing coding frame using *NVivo* 12.0 (QSR International, Doncaster, Australia). Codes were then grouped together based on similarities and differences forming a set of preliminary themes (descriptive themes). Some codes were dropped at this stage, as they could not be assigned to any groups. The research team considered the original research question and inferred the impact of the COVID-19 pandemic on families’ food decisions as it was captured by the descriptive themes. During this process a new set of final themes (analytical themes) were generated that directly addressed the study question. The creation of descriptive and analytical themes was supervised and agreed by the other co-authors.

## Results

**Fig. 1:**
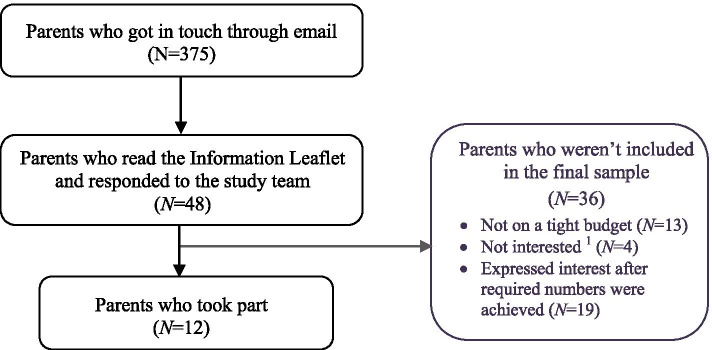
Participant selection flowchart. ^1^This can be further broken down to those who: didn’t have the time to take part in the interview (*N*=2); were not comfortable with being interviewed (*N*=1); didn’t give a reason (*N*=1)

A total of 375 parents got in touch through email to express their interest in finding out more about the study. The research team shared the Participant Information Leaflet with them and explained the purpose of the study and what participation entailed. From those who read the study information the first 12 parents who verbally confirmed living on a tight budget with a child 2–17 years were recruited in accordance with the stratification process. Figure 1 offers a detailed flowchart of the participant inclusion and exclusion process.

### Participant characteristics

All participants except one were the biological parents of the children in their household. One participant was the biological mother of two children and a stepmother to a further child. There was only one father in the sample. Several more eligible fathers expressed interest in the study, but only after their strata was complete and, thus, they were not selected. Participants were predominately white and looked after the family as their full-time occupation. Most families were receiving financial assistance from the government and had three or more children. Participants’ characteristics can be found in Table 1.

**Table 1:** Participant characteristics

	MEAN ± SD	RANGE
Children’s age (y)^a^	9 ± 4.5	2–15
Parents’ age (y)	38 ± 8.4	22–51
	***N***	**%**
Parents’ gender		
Females / Males	11 / 1	92 / 8
Number of children		
1 / 2 / 3 / 4	1 / 2 / 6 / 3	8.3 / 16.7 / 50 / 25
Ethnic background		
White / Asian	11 / 1	91.7 / 8.3
Country of birth		
Northern Ireland	7	58.3
England	3	25
Poland	1	8.3
Vietnam	1	8.3
Employment		
Employed full-time	1	8.3
Employed part-time	3	25
Home duties	7	58.3
Long-term sickness / disability	1	8.3
Location of residence		
Urban / Rural	9 / 3	75 / 25
Education level		
Secondary school	4	33.3
Non-degree qualification	3	25
University qualification	5	41.7
Eligibility for benefits		
Yes / No	9 / 3	75 / 25
Marital status		
Married or living with partner	6	50
Single	3	25
Separated	3	25

^a^Some of the participants had children outside the age range 2–17, which were not included in the descriptive statistics

The method of Photovoice was well understood among the participants and all of them provided photographs before their interview. There was a great variance in the number of photographs sent by the participants, which ranged from two to 17. The majority of participants’ photographs portrayed meals served for their family, followed by packaged foods purchased, food store prices and receipts.

### Study findings

**Fig. 2:**
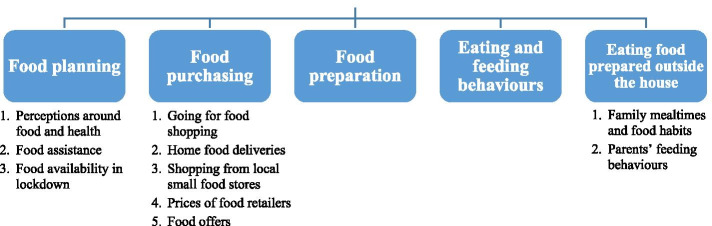
Themes and sub-themes as emerged from the study data

**Fig. 3:**
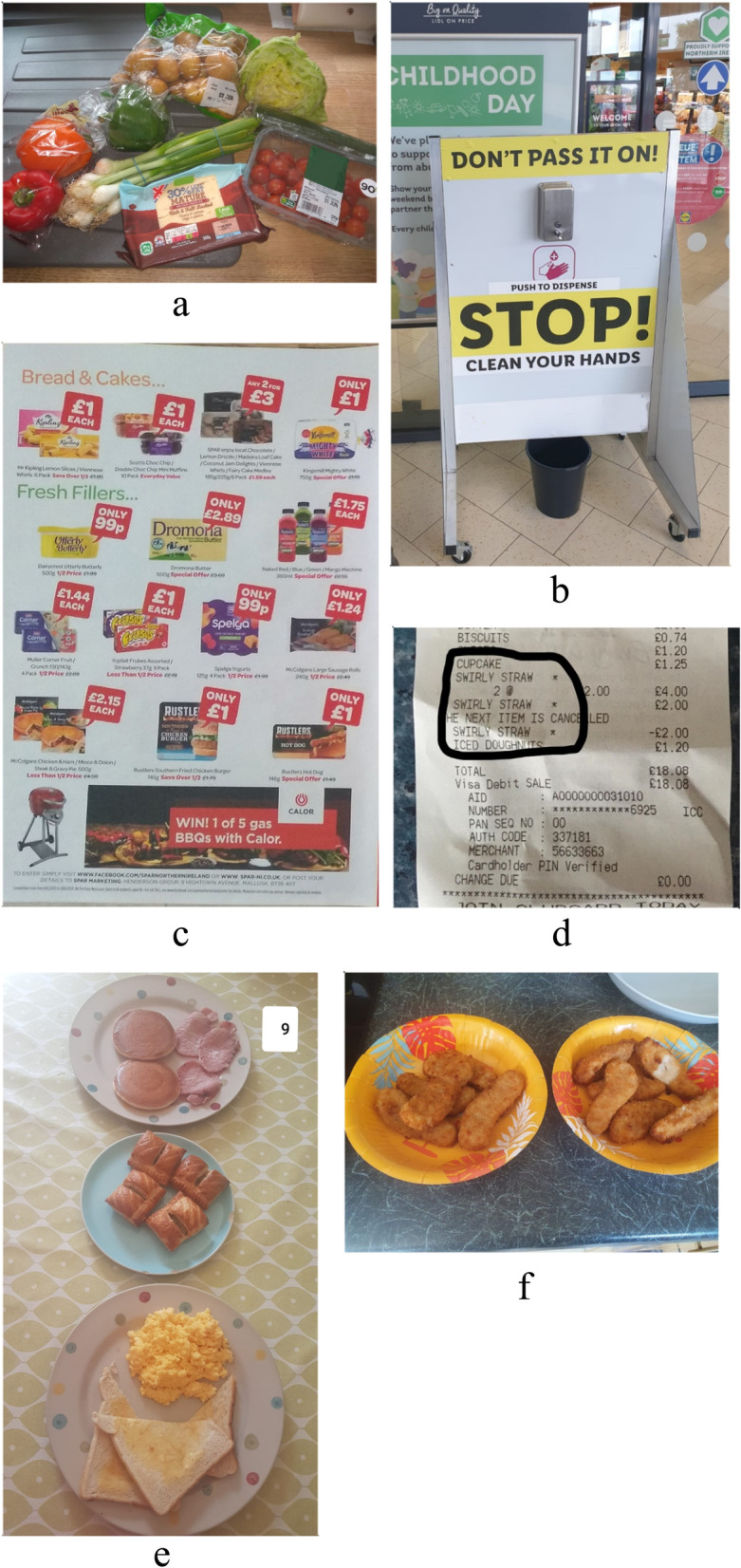
Participants’ photographs reflecting some of the sub-themes. **a**) Need to provide a nutritious diet. **b**) Store strategies to control the spread of COVID-19. **c**) Choosing foods on offer. **d**) Emotional snacking. **e**) Giving into children’s food requests. **f**) More convenient meals due to limited time

Five distinct themes were found within the data. Each one addresses a stage of the decision-making process in relation to food (planning, shopping, preparing, eating at home and eating outside the home) and discusses the impact of lockdown. A list of the themes and sub-themes, when applicable, can be seen in Fig. 2. Some allocated quotes can be seen in Table 2. A number of photographs that were discussed as part of the Photovoice exercise and reflect some of the main themes/subthemes can be seen in Fig. 3.

**Table 2:** Quotes voiced during focus groups for every theme and sub-theme

**Theme: Food planning**	
Sub-theme: Perceptions around food and health	
Need to provide a nutritious diet for growth and health	
*It is very important to me to try and get as much fruit and vegetables into this family every day, especially at the minute, think if anything I might be over-worried … I don't know if it's because of lockdown, I’m more aware of the family’s immune system and keeping them strong and robust…* (02 - Married mother of 3)	
*Every week I try get some like you know, different vitamins, the food have. Meat as well because meat has lots of protein in it, so they need like, children need them...* (07 – Married mother of 2)	
Basing food choice on child’s preference	
*I just see what the kids like and I'll keep doing the same thing…. As I say, for me it's just pure convenience and I know the kids will eat it.* (06 – Single father of 4)	
Sub-theme: Food assistance	
Help from family and friends	
*I have like three, three-four friends (who are) very kind, because they know I’m on my own most of the time. … This is, I think we are quite lucky, we’re very lucky to get help from friends with food.* (11 – Single mother of 3)	
*If we had anything spare, they (parents) just live around the corner from my house, we would give it round to them, or they would give it round to us. So we would help each other out too.* (03 - Married mother of 3)	
School meal assistance	
*I’d be lost without it. Ehm, I don't think it goes as far as the government maybe think it goes, but it’s definitely been very helpful*. (02 - Married mother of 3)	
*Fifteen pound a week wouldn’t really buy fruit for my children, you know? … But it’s definitely a brilliant help and I appreciate it a lot.* (10 – Married mother of 3)	
Help from the community	
*I actually every week, the local youth club help the likes of… me and my kids go to the youth club, the girls do. And they bring down like a wee, it's like a breakfast box for them. … It’s only a few wee odds and ends, but it helps out like. …. Especially when you're on a tight budget.* (09 – Single mother of 4)	
Sub-theme: Food availability in lockdown	
Concern over shortage of food	
*Like we couldn't get pasta and that’s, we would live on pasta, 'cause one of our dishes, we would have pasta, tuna and sweet corn as a dish every week. And we really couldn’t get pasta for ages. And we had family members looking out for pasta for us.* (09 – Single mother of 4)	
Engaging in panic buying	
*I sort of stocked up at the start as well, because I was panicking, you know. So I would have made sure… You know, like I bought extra of everything at the very beginning of this, because I was worried, you know…* (12 – Married mother of 3)	
Convincing the children of limited food availability	
*I would take them around shopping at our local Tesco’s and let them see that the shelves were bare, you know? That I wasn't just saying ‘oh I'm not making this, 'cause I don’t want to’…* (01 - Single mother of 3)	
**Theme: Food planning**	
Critical of bulk buying	
*I think people who have absolutely nothing, because they can't get things, they only need certain things and they can’t get them…because other people are bulk-buying them.* (01 - Single mother of 3)	
Importance of a well-stocked food cupboard	
*I have a well-stocked cupboard I suppose. I think I quite like having food in there that I could fall back on.* (08 – Single mother of 4)	
**Theme: Food purchasing**	
Sub-theme: Going for food shopping	
Shopping less often	
*…having to go out more often to get the fresh vegetables and that means being about people that potentially have COVID-19.* (02 - Married mother of 3)	
*Just everything is so organized, more organized now in my own house. … Plus like my food shop list is done before I would have never done a list and I would have shopped when... every fortnight, so just over 2 weeks. But now I'm doing it once a month just because I don’t want to go out as much as well.* (03 - Married mother of 3)	
Scheduling shopping journey to avoid queues	
*I tried to go first thing in the morning, when it’s not too busy. I kinda know now whenever not to go…* (09 – Single mother of 4)	
Unfortunate experiences in stores	
*I went to go a few times in the lockdown and the queue was right down the street and I just thought ‘no, I don’t wanna do it’. And the one time I did go in, there was people bending over you to get things, when you were really, really afraid! I never went back.* (10 – Married mother of 3)	
Store strategies to control the spread seen favourably	
*They have somebody standing at the door and they were saying ‘nobody else in’ and had all the restrictions in place, you know lines and things and indicators on the floor. So it was really well-prepped. I felt comfortable going in there.* (10 – Married mother of 3)	
Sub-theme: Home food deliveries	
Shift towards home food deliveries	
*I mean, before lockdown, I would have been using my local shops more, but obviously, because of lockdown I was trying to get stuff delivered and not be going out to the shops as often.* (12 – Married mother of 3)	
Difficulty in finding delivery slots	
*What had happened at the start of lockdown was that home delivery wasn't available, because so many people were using it. So you would have maybe had to book a fortnight in advance* (04 - Married mother of 2)	
Incomplete food deliveries	
*I have done, I would say six or seven orders now between the major chains and not once out of those six or seven orders have I got everything that I have ordered…. So I was making something specific, but then you’d open up your shopping and you’d be like ‘oh where’s the… where’s the carrots that I ordered?’, ‘where’s the mince steak that I ordered?’.* (01 - Single mother of 3)	
**Theme: Food purchasing**	
Sub-theme: Shopping from local small food stores	
Avoiding local shops	
*I used to go the bakery twice a week. ... but the queues to get into the bakery is just ridiculous, I'm not wanting to go and stand for an hour just to buy 6 hot-cross buns …* (03 - Married mother of 3)	
*Before lockdown I would have been using my local shops more, but obviously, because of lockdown I was trying to get stuff delivered and not be going out to the shops as often.* (12 – Married mother of 3)	
Feeling supported by local shops	
*I mean there was delivery services that you could've got, you know, during the time, like if I was sick or you couldn't go out. … I got that little fruit and veg shop to deliver to me. And then like a local shop could have sent me, I don't know, bread, milk and cigarettes, if I wanted.* (10 – Married mother of 3)	
Sub-theme: Prices of food retailers	
Observing prices going up	
*At the start now, they’ve only started going back on, they took all the offers off, you know the likes of ‘three for two’s’ or ‘buy one, get one free’. They all went off, so they did. And all the offers went off and I noticed all the prices did go up a wee bit.* (09 – Single mother of 4)	
*I've noticed a big change in prices at the minute.… I’ll do a shop monthly for my cupboards and I was like… and it usually cost me about 80 pounds for the whole month. And it’s gone up to for about 120, just everything has just been up 20, 30, 40 pence… I know it's not much in a way, but like it is much whenever you're not working*… (03 - Married mother of 3)	
Understanding for food retailers	
*I understand that firms have more outgoings at the minute than they have probably incomings, so they do have to put their prices up and balance it up a little bit.* (01 - Single mother of 3)	
Food prices going unnoticed	
*I didn’t actually see prices going up, but I’m not the best one to look at prices.* (10 – Married mother of 3)	
Sub-theme: Food offers	
Always choosing foods on offer	
*If there's something on offer you would buy it and that would be another idea for something to eat, you know for a meal. Whereas if there are no offers, you sometimes go ‘oh god, what should I buy then?’*. (08 – Single mother of 4)	
*It's 'cause I’m on my own with kids so I always would scrimp and save. If there’s something on offer, then I have to buy. I always budget when I'm shopping.* (09 – Single mother of 4)	
Importance of food offers increasing during lockdown	
*…I'm not at work anymore. We would go more to now look at like yellow labelled stuff. … Yeah, we would go to that section a lot more now than what we would have beforehand…* (03 - Married mother of 3)	
*Basically in the Covid, I haven't got a job anymore, so I had to cut corners a little bit. So I'm looking more for like special offers and then just introducing them to the family.* (10 – Married mother of 3)	
**Theme: Food preparation**	
Cooking more and healthier	
*I'm sort of glad that lockdowns been put on, if you know what I mean, because now that I'm cooking more than I've being a wee bit more healthier. Before lockdown I wasn't being healthy I was sort of relying on takeaways.* (05 - Married mother of 1)	
*… probably cooking a bit more just because I can cook things… When I was working, I might have called into Sainsbury’s on the way home from work and picked up a pizza or something, picked up something that was already made…* (08 – Single mother of 4)	
More convenient meals due to limited time	
*[I’m] single dad with four kids, so I would normally… with cooking I would normally do sort of chicken curries or spaghetti Bolognese, but during COVID there’s been a lot more ready meals, just because sometimes I would chill out with the children.* (06 – Single father of 4)	
Children getting involved in the kitchen	
*They’re staying up late, so what I find in the morning, my kitchen looks like Beirut… They will be making themselves a ready-made cheeseburger or hamburger.* (06 – Single father of 4)	
*And [child’s name], the 12-year-old, I've noticed she would come in as well and take an interest and cut things and will help... in the kitchen with the cutting and things and even stirring the chicken and making the curry up and things like that.* (09 – Single mother of 4)	
**Theme: Eating and feeding behaviours**	
Sub-theme: Family mealtimes and food habits	
Lack of meal structure	
*When you're in work, you’re kind of ‘right, people are going for their lunch’, you’re going, you’re going to sit down and have some lunch. You’re bringing something in for lunch, whereas now I have my breakfast… and then, I might be having my tea earlier.* (08 – Single mother of 4)	
*It’s (dinner) all over the place. Generally, it’s between 9 and 10, whereas before they probably would have been between 6 and 7.* (06 – Single father of 4)	
Emotional snacking	
*I think, for more as the weeks go by, we’re more fed up and… It's not a nice kept-up-in-the-house feeling. More and more everyone in the house will go on “is there any chocolate?”.* (02 - Married mother of 3)	
*They’re probably eating more rubbish, trying to eat more rubbish. But I think that’s out of pure boredom… if they were at school, they wouldn’t be eating*… (09 – Single mother of 4)	
Reduced fruit and vegetables due to infrequent shopping trips	
*I kind of suffered in the lockdown, because I would normally go, you know, within three days, two to three days and get fruit and vegetables, cause my kids would eat a lot of fruit. And I didn’t go, because I only wanted to go once a week, I was afraid of going out.* (10 – Married mother of 3)	
More family meals	
*Before (lockdown) we had no time to do that (family meals), because we just… my husband finish work late, I finished as well very late, sometimes so was different.* (07 – Married mother of 2)	
Sub-theme: Parents’ feeding behaviours	
Giving into children’s food requests	
*My 15-year-old stepdaughter…she's doing a lot of home schoolwork for GCSE’s. … she asked me to get Coke to keep just for her and to be honest that's the only reason I bought it. I feel sorry for her at the minute, because she is stuck in the house and it’s something just like a wee comfort thing to cheer her up…* (02 - Married mother of 3)	
Following a lenient parenting style	
*I will give them £5 each a day and I say ‘right, as long as you’re two meters from everyone else, enjoy life’ … ‘your mental health is more important at the minute and so is mine’.* (06 – Single father of 4)	
Dealing with children’s food refusals	
*…my wee ones are so young they’re like playing “I don't want eat that, I don't like that” like they would have only eaten sausages or chicken goujons … but now they’re just told to eat what's in front of them, otherwise they don't get anything until the next meal.* (03 - Married mother of 3)	
*I'm trying to get my son used to different textures of food… [Child's name] is funny with different textures and different feelings. He's got a touch of autism and he's got sensory issues…. so I try, I made a new dinner to see if he would eat it.* (05 - Married mother of 1)	
Making sure children eat enough	
*Cause I can cook food and if they don’t eat it, I will try to feed, feed them myself. I know I’m not supposed to feed them, but I just would like them to get something to eat, so that’s why… But now because I say ‘oh they’re at home now’ so we have more time so I just cook and try to offer food.* (11 – Single mother of 3)	
**Theme: Eating food prepared outside the house**	
Take-away as a reward	
*So it's been intense with the amount of (school)work that's been thrown at them and the amount of stuff that they have been expected to do without really knowing it. So Friday is our day to just let off a little bit of steam, so we would get a takeaway of some description.* (01 - Single mother of 3)	
Ordering affordable take-aways	
*I would look at Deliveroo, Just Eat and Uber Eats and I would look to see if there was a deal. And I would use all those services, when I found certain deals…. I'm generally ordering the same things once I knew the children were eating them.* (06 – Single father of 4)	
Reducing take-aways saved money	
*The takeaways are quite dear like if you go seven days a week with them. But now that I’m cooking, it's not really, so we have sort of saved quite a bit of money.* (05 - Married mother of 1)	
Sceptical about safety of food prepared outside the house	
*At the minute, I’m not sure, because it’s other people prepping food. I'd rather, it's safer just doing our own. … (I’m) not even sure about using all of them (takeaways) again. I don't know who's touching our food now and all this virus thing and… I’m trying to keep my kids safe.* (09 – Single mother of 4)

#### Food planning

This theme discusses participants’ considerations when deciding the type and amount of foods they purchased for their family during the first lockdown. Diverse thoughts were voiced, concerning participants’ perceptions around diet and wellbeing, food-related support and food availability during the period of lockdown.

##### Perceptions around food and health

Most parents explained that ensuring a healthy diet for their family was a priority. Their food planning aimed to provide an all-inclusive balanced diet which would keep their children and, when applicable, their partner strong and healthy. Fruit and vegetables were, for some of them, an important component of a healthy diet too and therefore, they always featured in their food shopping lists. In contrast, a few parents voiced that the choice of foods was mainly based around their children’s preferences.

##### Food assistance

Food-related support, such as food donations and help with food shopping, was stated to be crucial, especially among single parents. During the lockdown period in particular, food assistance offered by friends and extended family members was much appreciated, given the financial constraints experienced by the majority of families.

As part of discussions around food-related help, school meal assistance was also addressed. Most families that took part had children who were eligible for free school meals and, while schools were closed, they received financial help in the form of a biweekly bank transfer. Although all parents agreed that this initiative was extremely helpful, they also acknowledged that their family’s food needs had increased since they were staying indoors and the additional money was not sufficient to cover the increased requirements. Two families reported they received a box of food items from their local youth club on a weekly basis. This help was also welcomed.

##### Food availability in lockdown

A number of participants touched on the reduced variety of food products in supermarkets at the beginning of lockdown. The majority of them explained that the reduced food availability did not affect their families’ diet. On the other hand, some admitted that shortage of food was a major concern of theirs and few even engaged in panic-buying. Few participants recounted that their children had to do without some of their favourite meals due to the low food availability and closure of restaurants. Two mothers felt that their children had to witness the closed food outlets and limited stock on supermarket shelves to grasp the situation.

One participant criticised panic-buying behaviours and highlighted the need to be considerate of people who cannot access food due to limited availability. Two participants however emphasised the value of maintaining a well-stocked pantry at all times, irrespective of lockdown.

#### Food purchasing

Participants went into great detail when talking about their food shopping habits and their experiences in stores (or online shopping). Food cost and price reductions since the start of COVID-19 restrictions were also discussed.

##### Going food shopping

The outbreak of COVID-19 and the relevant restrictions had a major impact on parents’ shopping habits. For those who kept going to the supermarket, as opposed to having food delivered, the frequency and duration of shopping trips decreased because of fear of being exposed to the virus. Parents explained that more planning went into food shopping to ensure that their food basket covered their family’s dietary requirements for longer.

Visiting food outlets less often had an impact on the amount of fresh food products purchased. One mother pointed out that her family’s consumption of fresh fruit and vegetables decreased due to her infrequent shopping trips. Similarly, a mother admitted that the reduced access to fresh vegetables prevented her from cooking healthy meals as part of her weight management programme.

In terms of choosing supermarket or when to go shopping, the length of queues was considered to be a determining factor. Participants observed that some of their local supermarkets were very busy which motivated them to seek an alternative food shopping route or to schedule their shopping trip at less busy times. Unfortunate experiences at store queues or within stores were described to give further rise to fear when shopping.

A different perspective was expressed by two mothers. Their shopping trips and experiences did not change throughout lockdown and they maintained the same frequency of visiting the food stores. One of them shared that the need to feed four children gave her no other choice.

Furthermore, participants observed certain adaptations in the environment of supermarkets in line with social distancing and safety measurements. These included placing hand-sanitizers and employing strategies to avoid over-congregating, such as monitoring the number of people that enter the shop. Such adaptations were reported to increase confidence in shopping.

##### Home food deliveries

Online food shopping and home deliveries were extensively discussed in the interviews. A number of supermarket chains provided the option for individuals to go online and order food items. Several mothers talked about taking advantage of this service in an effort to minimise their trips outdoors. One mother, who had already been familiar with supermarket food delivery services, described them as a ‘necessity’ during lockdown.

Despite the convenience of home food deliveries, a number of problems were reported. To use the service, customers had to book a delivery slot first which, according to participants’ experiences, would invariably be 2–3 weeks away. As a result, effort went into assembling a shopping basket that contained food items that would last until the next order comes within a few weeks. In certain cases, participants admitted they could not use the service, as all delivery slots were booked out. Participants explained they also had to deal with incomplete food deliveries. As a consequence of long waiting times and limited supermarket stocks, food orders reached customers without certain items. Furthermore, relying on specific supermarket chains to get foods delivered meant that customers had to accept their prices, even though they knew that other supermarkets had more competitive prices in some products. One mother observed that the cost of the food delivery service was also a barrier, as she noticed a two-fold increase in the price over lockdown (from £3 to £6).

##### Shopping from local small food stores

Participants talked about the food shops they had in their local area and their role in food shopping since the COVID-19 restrictions were put in place. Conversations revealed mixed feelings in relation to shopping from these stores.

After the re-opening of local businesses, some of the stores could accommodate a certain number of people at a time and, hence, waiting time to get in increased. Similarly to the situation with supermarkets, participants explained that the long queues formed outside prevented them from shopping in these stores, even if they used to in the past. In terms of buying meat, it was generally agreed that the quality of meat products purchased from the local butcher was higher compared to those available in supermarket. However, due to long queues, along with supermarkets’ lower prices, participants opted for supermarket meat rather than from the local butcher. Moreover, there were also parents who avoided visiting shops overall and relied exclusively on food deliveries.

On the other hand, there were mothers who believed that local stores were important in the COVID-19 era, particularly for families that lived far from big supermarkets and did not want to travel far to purchase food items that their family needed on a daily basis. Some also conveyed that food products locally available were of better quality and, as restrictions eased and store queues became shorter, they intended to shop more often from small shops in the neighbourhood. A few mothers felt more supported by the local businesses than big supermarkets, as their local stores offered home food deliveries that reached families quicker compared to supermarket chains.

##### Price of food retailers

A few participants highlighted that supermarkets had increased the prices of food products and that the number of food offers was reduced since the restrictions were put in place. Some parents expressed frustration over the additional cost of their habitual shopping basket, as this was a difficult time for their families’ finances. However, one mother showed understanding of the circumstances of food retailers who had to increase their prices to make up for their increased expenses. Two women admitted that they had not noticed any difference in the price of food retailers ever since lockdown restrictions were enforced.

##### Food offers

Food offers was a topic that repeatedly came up in the discussions. Deals and reduced-to-clear items seemed to be a part of families’ diet before, as well as during the pandemic. A tendency to stock up on regularly bought foods, when they were on offer, was also reported.

Particularly since lockdown the difficult financial circumstances had given food offers a more prominent place in families’ shopping priorities. Parents used the extra time due to furlough or loss of work to time their shopping when the daily stock clearance took place. One mother explained that the reliance on reduced-to-clear foods prevented her from ordering home food deliveries, even though she acknowledged the safety of having food delivered at home during the pandemic.

#### Food preparation

Lockdown seemed to have a positive impact on cooking, as most interviews showed. Parents who had been furloughed or worked from home talked about the additional available time which urged them to cook more from basic ingredients, as well as to plan and to try more time-consuming recipes. Cooking home-made foods was also encouraged by the closure of take-away restaurants and the limited access to ready-made foods and snacks.

On the other hand, lockdown did not provide everyone with more time to cook. A single father of four admitted that having all children at home limited his available time and as a result, he resorted more to ready-made meals.

During lockdown, some of the parents, mostly those with older children, described that their children were becoming more involved in the meal preparation, such as heating ready-made meals for themselves or helping their parents by assisting in the kitchen e.g. chopping. This often led to increased mess in the kitchen.

#### Eating and feeding behaviours

This theme addresses changes in families’ eating habits and parental feeding practices, as outlined and experienced by the participants during the course of lockdown.

##### Family mealtimes and food habits

Interviews revealed several changes in families’ food habits. Parents who did not go to work during lockdown discussed the lack of structure within their day with one of them recounting skipping main meals as a result of it. The timing of meals was different too, as children did not go to school and followed an unpredictable daily routine.

Home schooling and staying at home seemed to encourage consuming more food in the form of snacking and this was voiced by the majority of participants. Snacks were mostly described to be confectionary and high-sugar drinks. Feelings of boredom and of ‘being stuck’ triggered by staying at home were quoted by a few of the participants as the reasons for increased snacking.

Apart from the higher consumption of snacks, families’ intake in other food groups seemed to remain roughly the same throughout lockdown. One mother however, mentioned consuming lower amounts of fresh fruit and vegetables because she was not going as often for grocery shopping. The same participant also highlighted that her family’s meat consumption had decreased since the COVID-19 outbreak, as she avoided going to her local butcher and supermarket’s meat was not of the same quality and taste. Minimizing the number and frequency of shopping journeys were the reasons for both changes. For some parents staying at home meant that they had more time to sit down with their families and share a meal.

##### Parents’ feeding behaviours

During lockdown some parents explained that they had to engage in certain feeding behaviours that varied from those employed in the past. One of them was giving in to children’s food requests to placate them under the unusual circumstances of lockdown. It was pointed out that children would ask for snacks or their favourite meals more frequently as a result of being at home longer or being pressured by schoolwork assigned online. This often seemed to go hand in hand with following a more flexible and lenient parenting style overall.

On the other hand, a few mothers mentioned that the additional time spent at home and closure of fast-food outlets offered a window of opportunity to introduce them to new eating experiences and deal with food refusals. One mother expressed concern over her daughters’ low body weight and explained that this motivated her to take advantage of the extra time to make sure they ate enough.

#### Eating food prepared outside the house

Most families bought food prepared outside the house prior to COVID-19 restrictions. Eating-out habits involved visiting fast food restaurants and ordering takeaways with a frequency varying from once a week/fortnight to regularly within the week. Some families maintained the habit of ordering takeaways during the course of lockdown and sometimes this was described as a reward for the family being stuck at home or children doing schoolwork. Two mothers mentioned that they continued ordering from their local takeaways in an effort to support local businesses. Ordering affordable take-away meals was particularly important for a single father during lockdown, since they kept all his four children pleased.

Several families on the other hand, discussed how they had to cut down on their fast food and takeaway intake due to closure of their local restaurants. A few parents saw it as an opportunity to reduce their unhealthy eating-out habits and to start cooking more. This situation also benefited families’ finances, as ordering food cost them significantly more than cooking meals at home, as parents came to realise. This was particularly evident for the families who heavily relied on take-away foods in the past. Takeaways were not part of family’s regular diet in three households, so their limited availability due to lockdown had no impact on their dietary habits.

As restrictions on food restaurants were easing during the course of the interviews, it was pointed out that the main fast food chains were re-opening and this was met with great enthusiasm from the children. However, the long queues outside the restaurants often discouraged parents from purchasing food from there. One mother talked about the importance of being alert and mindful of COVID-19-related restrictions. She expressed limited trust in the safety of food provided by restaurants which led to reluctance in ordering food for her family from outside.

## Discussion

This qualitative study presents parental perspectives of the impact of COVID-19 lockdown on food decisions among low-income households. The restrictions put in place to inhibit the spread of the virus had an impact on all aspects of dietary-related practices from food planning and shopping to cooking and eating. The following paragraphs discuss the main diet-related changes as these were described by the study participants.

Parents’ desire to provide their families with a nutritious and balanced diet, in conjunction with the additional time available throughout lockdown, encouraged the preparation of home-made meals. Additionally, the closure of restaurants and fast food chains, as well as the realisation that infrequent takeaways helped with family expenses, further contributed to the increased intention to cook from raw ingredients. This is in agreement with survey data from the island of Ireland, Great Britain and Italy showing that individuals cooked and consumed more in-home meals in the lockdown period than prior [20, 21, 36]. Pre-COVID qualitative explorations with low-income parents have shown that in the UK lack of time was a barrier to cooking from raw ingredients [37, 38]. Additionally, issues of peer pressure among children, particularly older children, to consume fast food have also been highlighted [37, 39]. In this sample, lockdown eliminated both of these barriers, i.e. lack of time (for some parents) and peer influence, leading to more home-made meals. This is undoubtedly a positive consequence of lockdown, as consumption of home-made meals is associated with better nutritional outcomes compared to ready-made food. Previous research suggests that individuals who cook frequently are more likely to follow healthy dietary patterns and to meet the recommendation of fruit and vegetable consumption, when compared with those who frequently consume ready-made foods [40–42]. On the contrary, diets characterised by frequent consumption of fast food and ready-made meals are associated with increased caloric intake that can, in the long term, contribute to positive energy balance and overweight [43, 44].

On the other hand, an increase in unhealthy snacking was reported for both parents and children. Frequent consumption of confectionary and sugar-sweetened drinks was self-justified, because they were seen as a reward for intense school work or a way to avoid boredom and calm stress and other negative feelings triggered by isolating at home. High consumption of unhealthy sweet and savoury snacks was also seen among overweight and normal weight adults in Italy, Poland and Ireland, as recorded by observational data [45–47]. This trend in snacking behaviours was also seen in a study that surveyed 800 respondents in the Unites States in June 2020, where emotional eating triggered by stress during the first COVID-19 was associated with choosing foods based on convenience and sensory appeal [48] This shift in eating habits is not surprising, as previous research work suggests that individuals are more likely to resort to comfort foods, when they experience negative emotions and/or they feel the need to reward themselves [49]. Increased desire for high-sugar and saturated foods can lead to increased energy intake paired with low intake of essential nutrients, which holds long-term health and weight implications [50, 51] and therefore, should be at the forefront of dietary recommendations during and after the COVID-19 era.

Although levels of concern for virus transmission risk seemed to widely vary, parents generally adhered to the COVID-19 restrictions and conveyed reluctance to leave the house, which determined to a large extend their food shopping patterns. On one hand, fewer shopping trips and longer waiting times for food deliveries motivated parents to plan their families’ food demands more efficiently. Better food planning during lockdown, also seen in another UK report of qualitative data [52], could be established as a habit that over time can have further positive implications e.g. avoiding impulse buying, reducing food waste. However, infrequent shopping trips and reliance on supermarket home deliveries were also presented to compromise home food availability in fresh food products (e.g. fruit, vegetables and fresh meat) with potentially negative effects on dietary quality. Data from a low-income and vulnerable population living in England also highlighted challenges associated with trips to the supermarket and securing good food availability with online shopping [36]. The effect of these challenges on families’ dietary intake is yet to be fully understood and requires further investigation.

Financial constraints experienced by low-income families in this study were exacerbated during lockdown by loss of work and, in some cases, by perceived elevation in supermarket prices. Indeed, survey data from Northern Ireland indicate that during the course of the first lockdown 18% of households experienced an income decrease of 24% or higher and that the proportion of people experiencing symptoms of food poverty reached 20%; a proportion that was 8% before the COVID-19 pandemic [53]. In terms of food cost, analysis of the average UK grocery prices shows a price increase shortly after lockdown was enforced which persisted in the following weeks [54]. Rise in food prices alongside the cost and perishability of healthy foods (e.g. fruit and vegetables), with the latter having already had a negative impact on food shopping choices of low-income parents in the UK [37–39], led parents from this sample to search for ways to budget when food shopping. As a result, food offers and reduced-to-clear items had a prominent place in families’ shopping basket. At the same time, the school meals payment scheme and food donations were perceived to lessen families’ expenditure on food and to considerably contribute towards their food security. This finding is not surprising at a time where food banks across the UK saw a rise in demand and low-income families were most in need of them [55]. Additionally, it is debatable whether increased reliance on reduced-to-clear foods can secure dietary diversity for the family meals and whether it raises concerns around food safety, especially if appropriate food storage methods are not applied.

Present findings indicate that families experienced lockdown in distinct ways depending on their type and size, which seemed to influence the way family members modified their food behaviours. For instance, even though lockdown generally encouraged more cooking and shared family mealtimes, this was not the case for single parents with many children who were home-schooling, as parenting during lockdown did not seem to allow time for home-made food preparation. Instead, they sought more convenient food choices they knew their children like, such as ready-made meals. Distinct influences of COVID-19 on dietary behaviours of single-parent households have been previously highlighted in a survey of 13,829 Australians, which showed that those living with children and without a partner were particularly likely to report overeating [56]. Even though this type of influence did not emerge in the present paper, it indicates that single-parent families may have found it more challenging to adopt a healthy diet during the COVID-19 lockdown/s compared to parents living with partner. Among families with adolescents, parents observed their children’s independence in the kitchen and their ability to prepare basic meals with minimal adult supervision. Older children’s involvement with food preparation allowed parents more time to devote to younger children’s diets or to other parenting tasks. Additionally, although the level of funding for the school meal assistance was less than ideal, the scheme was a valuable help for all low-income households and, along with food donations seemed to be an essential help for single-parent families to remain food secure.

### Limitations

Although the study sample consisted of mostly white parents (apart from one Asian mother), participants came from diverse backgrounds in terms of nationality, location of residence (urban/rural) and age and number of children. Thus, it is envisaged that the study findings reflect the experiences of most parents of white ethnicity who live in economically disadvantaged households. Food insecurity was not an emerging theme in this study contrary to what was expected, considering that in the latest ‘Food and You’ survey 12% of households in Northern Ireland were classified as marginally food secure [57]. In addition, the sample was female-dominated and included only one father. This could be seen as an important limitation, as nowadays food decisions fall more within the sphere of responsibility of males than two decades ago [58]. However, studies in the UK and US highlight that within shared households a higher proportion of women is responsible for food shopping and preparation, and therefore, a female-dominated sample may offer a more accurate reflection of food decision-making in UK households [58, 59]. As part of the adaptations in research methods during the pandemic, all interviews took place online, as opposed to face-to-face. In this way, certain elements of in-person interactions (e.g. body language) that facilitate rapport and encourage probing were lost, which has been previously reported with online interviews [60]. Nevertheless, video-conferencing platforms have been widely used as part of work and home-schooling during the COVID-19 pandemic and all parents included in this study were familiar with using such technologies without reporting technical difficulties. Employment of web conferencing technologies also enabled us to engage with geographically dispersed individuals in urban and rural area in Northern Ireland. Finally, the participatory photo-elicitation method employed in this study added strength in the data collection process, as they encouraged a good level of involvement from all study participants and added important insights into nutrition-related challenges of low-income families, which may have been overlooked by a traditional semi-structured interview schedule. Participants photographed similar situations with very few of them diverting from the common topics. For example, only one mother sent us pictures of adjustments made by her local supermarket for COVID-19 and of her walk to the supermarket; and only one mother captured a take-away meal she had with her family. Despite the uniformity of topics across participants’ photographs, these photographs acted as triggers to narrate personal stories that shed light into their family’s food-related decisions. As previously highlighted in a systematic review on the use of the Photovoice in public health research [61], Photovoice is a flexible tool to generate photo-elicited discussions in relation to health and dietary behaviours. Moreover, its use is not restricted by participant characteristics (e.g. financial status or literacy level) [62].

## Conclusion

Overall, this qualitative study revealed that food decisions of low-income families were heavily affected during the period of COVID-19 lockdown. Parental perspectives voiced in this study generally align with changes in dietary behaviours observed across the UK, such as increased preparation and consumption of homemade food, as well as frequent unhealthy snacking. Store queues and long-awaited home food deliveries motivated good meal planning, but also made access to fresh and nutritious food difficult. Food donations offered by the closer and wider social circle, along with monetary assistance replacing school meals, proved to be crucial for parents who had to minimise cost and feed their family within a limited budget. This study adds to the growing body of research on dietary behaviours during the COVID-19 pandemic by providing an insight into the nutritional challenges experienced by low-income families. It also highlights that further food-related support is required from local and government-led initiatives to mitigate the increased risk of food insecurity among economically disadvantaged families.

## Supplementary Information

**Additional file 1.**

## Data Availability

The datasets used and/or analysed during the current study are available from the corresponding author on reasonable request.
